# Characteristics and treatments of ocular blast injury in Tianjin explosion in China

**DOI:** 10.1186/s12886-020-01448-3

**Published:** 2020-05-06

**Authors:** Yuanyuan Liu, Kang Feng, Hao Jiang, Fuhua Hu, Jun Gao, Wanhong Zhang, Wenjing Zhang, Bo Huang, Rodrigo Brant, Cheng Zhang, Hua Yan

**Affiliations:** 1 Department of Ophthalmology, Tianjin Medical University General Hospital, No. 154, Anshan Road, Tianjin, 300052 China; 2 Tianjin Neurological Institute, Key Laboratory of Post-Neuroinjury Neuro-repair and Regeneration in Central Nervous System, Ministry of Education and Tianjin City, Tianjin, China; 3 Department of Ophthalmology, Peking University Third Hospital, Beijing, China; 4 Department of Ophthalmology, Tianjin Eye Hospital, Tianjin, China; 5 Department of Ophthalmology, Tianjin Port Hospital, Tianjin, China; 6 Department of Ophthalmology, Tianjin Third Central Hospital, Tianjin, China; 7 Department of Ophthalmology, Tianjin TEDA Hospital, Tianjin, China; 8 Department of Ophthalmology, Affiliated Hospital of Logistic College of Chinese People’s Armed Police Forces, Tianjin, China; 9 Department of Ophthalmology, University of Mississippi Medical Center, Jackson, MS 39216 USA; 10 Department of Ophthalmology and Visual Sciences, Federal University of São Paulo, São Paulo, 04023-062 Brazil; 11 Department of Ophthalmology & Visual Sciences, Montefiore Medical Center, Albert Einstein College of Medicine, Bronx, NY 10469 USA

**Keywords:** Ocular blast injury, OTS, Globe injury, BCVA

## Abstract

**Background:**

To document characteristics and treatments of ocular blast injury from a fire and explosion.

**Method:**

**A**uthors retrospectively evaluated 116 patients with 166 eye injuries from six hospitals. Terminology of ocular injury referred to Birmingham Eye Trauma Terminology, and best-corrected visual acuity (BCVA) was categorized with the ocular trauma score (OTS) grading system. Incidence, preoperational and follow-up BCVA, treatment of severe ocular blast injuries were surveyed.

**Results:**

Oculoplastic injuries accounted for the majority of eye injuries, while globe injuries were presented in 52 eyes with median baseline OTS 70 ranging from 26 to 100. No endophthalmitis occurred. The mean timing of the first-stage operations was 9.4 ± 6.4 h after blast, while second-stage operations were performed on average 14.7 ± 0.9 days post blast. Final BCVA of 68.8% of eyes achieved 20/200 or better as followed, 7 open globe injuries had a BCVA of no light perception. Additionally, eyes presenting rupture, retinal detachment, vitreous hemorrhage, choroidal injury and initial BCVA less than 20/200 had worse final visual outcomes, while globe penetration was not associated with poor visual acuity.

**Conclusion:**

Various ocular injuries were commonly in the casualties of blast, in which open-globe injuries have worst visual prognosis. OTS is a valid approach for evaluation of prognosis and optimizing the therapeutic strategies subsequently in the massive casualty. Intense rescue and careful examination, proper surgery should be performed correctly to rescue patients.

## Background

On August 12, 2015, an unexpected explosion occurred in the container storage station at Tianjin Port, China at around 23:30, followed by a series of blast, in which the second blast is the heaviest one. According to the authoritative record, the main cause of the explosion was the reaction of stored hazardous chemicals [1]. Explosions causing mass casualties associated with war, bombing or terrorism have been documented in, the bombing in Oklahoma (1996), World Trade Center Terrorist attack (2001), the Iraq War (2003), the Boston Marathon bombing (2013), occurred around the world [2, 3]. According to the official news, there were 17,000 home and 170 business involved in the explosion, 114 individuals died and 722 individuals sent to the hospital for the therapies of the injury caused by the Tianjin blast [4]. Within hours, the local hospitals activated the disaster response for the blast to implement the treatment of injuries. Casualties suffered included blunt or sharp trauma which were examined by the ophthalmologist in the emergency room, including Computed Tomography, Magnetic Resonance Imaging where necessary.

Injuries resulting from explosions are classified into four parts [5, 6]. Firstly, the detonation wave itself can cause the primary injury to some extent; Secondly, fragments propelled by the explosion, like glass, dust, masonry from some constructions damaged; Thirdly, acceleration of body resulting from blast wind caused displacement on victims; Finally, the tremendous and temporary heat produced by the explosion results in some thermal injuries. Based on the distance to the explosion, severity and source of the explosion, the types of the injuries vary [7]. Ocular injury occurs at a high incidence in the terrorist blast victims due to the eye superficial exposure [8].

Ocular injuries associated with combat and terrorism targeting the civilian or military have been reported, however, a study of ocular injuries associated with a chemical explosion has not been published. In this study, we retrospectively analyzed the different categories of ocular blast injuries with certain outcomes collected by 6 major hospitals attending the rescue, and all patients were treated efficiently after the blasts. The aim of this study is to describe the category, cause, visual outcomes, and treatment of the ocular injuries of survivors in the Tianjin port blast and attempt to optimize a proper and efficient approach to provide precise treatment to the similar explosive casualties, especially for the ophthalmologists.

## Methods

The study conformed to the requirements of the Declaration of Helsinki, and informed consent form was obtained from all subjects recruited in this study. This study was also approved by Tianjin Medical University medical ethics committee.

The authors retrospectively collected information about patients who suffered ocular and adnexal injury in Tianjin Port explosion on August 12th. All patients were identified from inpatient records, outpatient records, emergency records, surgery reports and all existing data of each patient registered in six main hospitals in Tianjin, i.e. Taida Hospital, Tanggu Hospital, Tianjin Medical University General Hospital, Tianjin Eye Hospital, Tianjin Third General Hospital, and Tianjin Armed police Hospital. Patients were excluded where injuries were unrelated or indirect to explosive events during the bombing, including patients coming from the detonation sites.

Emergency surgeries were operated depending on the patients’ conditions, and the eye injuries cannot be recognized as priority considering saving patients’ lives promptly. In order to record the thorough details about the ocular injuries from the initial examination to the end of the follow-up, the form was designed including the following variables: demographic information of the patients (name, gender, address, and age), injury location, eye condition, associated systemic injury, treatment, post-treated condition. Injuries were classified into open and closed global injuries in accordance with the Birmingham Eye Trauma Terminology (BETT) [9], while ocular plastic or neuro-ophthalmologic injuries were classified as injuries without global involved (Table 1). Ocular foreign bodies, orbital fracture and traumatic optic neuropathy were confirmed both by the direct visualization and the radiologic imaging. Multiple injuries were recorded such as multiple corneal foreign body, eye lid laceration complicated with open global injury.

**Table 1:** Classification of ocular blast injuries with numbers

Ocular plastic injury (114 eyes)	Eyelid injury only (75 eyes)
	Eyelid injury combined with globe injury (28 eyes)
	Orbital fracture (14 eyes)
	Lacrimal injury (2 eyes)
Closed globe injury	Ocular Contusion (Zone I and II) (8 eyes)
	Ocular Contusion (Zone III) (19 eyes)
	Lamellar laceration (4 eyes)
Open globe injury	Ocular rupture (6 eyes)
	Ocular penetration (15 eyes)
	IOFB (1 eye)
Neuro-ophthalmological injury	8 eyes

Classification of the ocular blast injuries were made according to the BETT.IOFB=Intraocular foreign body

Ocular Trauma Score (OTS) was developed to predict the outcomes of visual acuity in ocular injuries efficiently by Kuhn and his colleague, in which the higher OTS demonstrated a better prognosis on the visual acuity [10]. In this study, we used the data from medical documents to calculate the OTS retrospectively for statistical analysis. The best corrected visual acuity (BCVA) was categorized as the following 5 grades: no light perception, Light perception (LP)/Hand Motions (HM), 1/200 to 19/200, 20/200–20/50, 20/40–20/15, and the final BCVA was documented during the last follow-up. OTS was performed in the 52 eyes of 43 patients defined as global injuries, and the eyes were graded into 5 categories referred to in Table 2: category 1 (0 ~ 44), category 2 (45 ~ 65), category 3 (66 ~ 80), category 4 (81 ~ 91), category 5 (91 ~ 100). In order to explore the risk factors of prognosis, univariate and multivariate logistic regression analysis was performed in patients who suffered global injuries with BCVA above 20/200 compared with BCVA below 20/200 and recorded as odds ratios (OR) with 95% confidence intervals (CI).

**Table 2:** Calculation of Ocular Trauma Score (OTS)

	Variables Used	Raw points
A	Initial vision	
	NLP	60
	LP/HM	70
	1/200–19/200	80
	20/200–20/50	90
	≥20/40	100
B	Perforating injury	−14
C	Retinal Detachment	−11
D	APD	−10
E	Rupture	−23
F	Endophthalmitis	−17

Table Calculating the sum of the raw points: A + B + C + D + E + F. APD: afferent pupillary defect; *HM* hand move, *LP* light perception, *NLP* no light perception

## Results

**Fig. 1:**
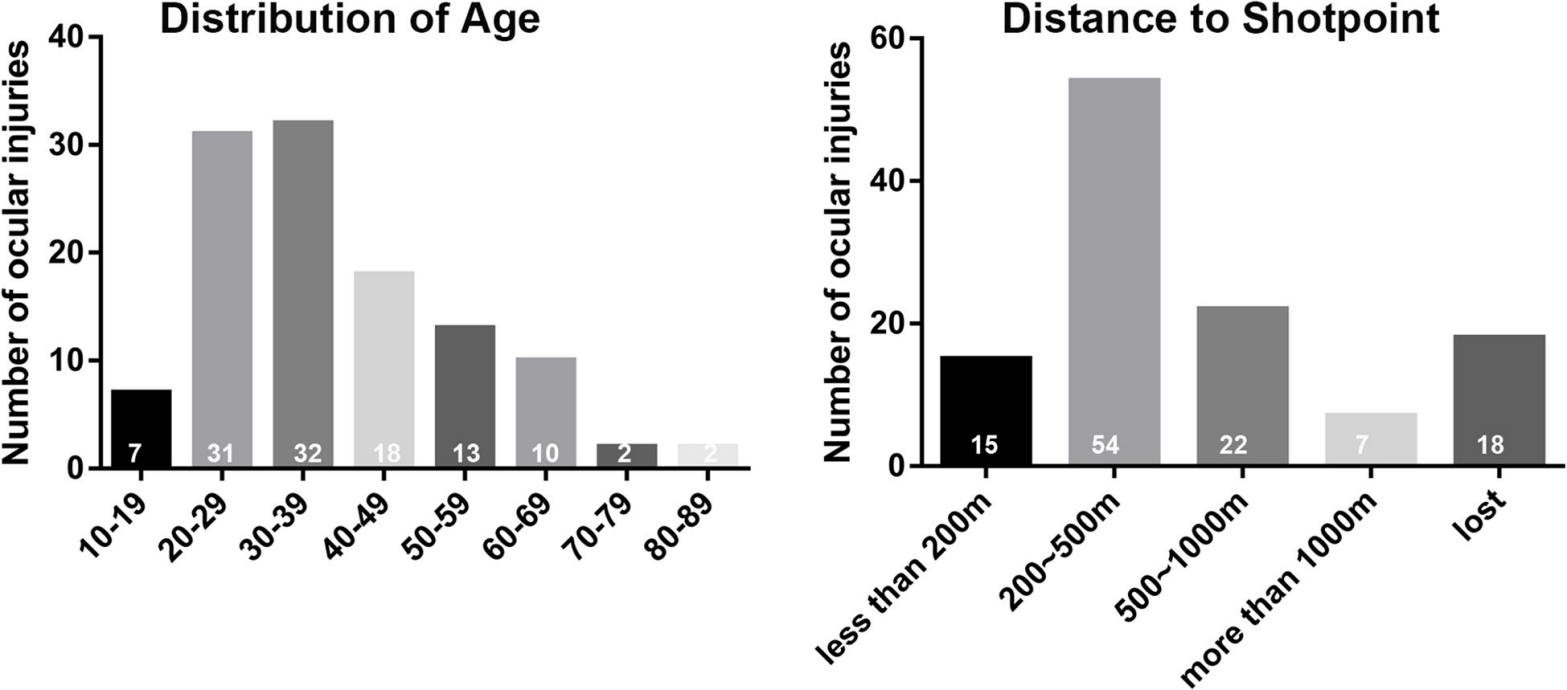
Age distribution of victims in the blast with the distance to the shotpoint when injuries occurred. Ages of most of the victims were ranged from 20 to 39 years old. The majority of the victims were 200 ~ 500 m from the shotpoint when the explosion occurred

After the explosion, most of the patients were immediately transported to the hospitals nearby, where they were triaged to the other hospitals located far from the explosion sites. The biographic characteristics of ocular injury were illustrated in Table 3. There were 116 survivors (88 males, 28 females) who suffered ocular injuries in the explosion involved in this study in total. The age of the patients ranged from 15 to 82 years old, and the median of the age was 35 years shown in Fig. 1. For patients who provided the exact injury location, home was the main injury location (58.6%), next was outside (i.e on driving, on duty, walking on the road) with 13.8%, while 12.1% were on the scene of the explosion (most of them are firemen who attended the rescue of the explosion). Most specifically, 59.5% of the ocular injuries occurred within 500 m of the explosion site, 19% were within 1000 m, and 6.1% were out 1000 m illustrated in Fig. 1.

**Table 3:** Characters of Ocular injuries in Tianjin explosion

Gender	88 male 28 female (3:1)
Age	Median 35 years (range 12–82 years)
Unilateral/Bilateral	73/43
Injury patterns	Globe injury (52/159 eyes)
	Oculoplastic injury (114/159 eyes)
	Neuro-ophthalmologic injury (8/159 eyes)
Associated systemic injury	40/116
Mechanism of injury	Glass (68/116)
	Wave of blast (10/116)
	Debris of blast (8/116)
Documented timing for surgeries (post explosion)	First-stage surgery 9.4 ± 6.4 h (range, 1 ~ 48 h)
	Second-stage surgery 14.7 ± 0.9 days
Location of victims	Home (68/116)
	Outside (16/116)
	On the scene (14/116)
Distance to shotpoint	Less than 500 m (69/116)
	500 ~ 1000 m (22/116)
	More than 1000 m (7/116)

There are 58.6% of the injuries were due to the blasts and fragments resulting from the explosion with the proportion of 58.6%, glass shards were the most common fragments. Of 65.5% victims were isolated ocular injuries, while 34.5% ones with various degrees of concomitant systemic injuries; of the 159 ocular injuries, 64.8% ones were ocular adnexal injuries individually, 32.7% cases were globe injuries singly, and 17.6% victims were combination of the above.

All patients were treated in the emergency department or routine outpatient room and hospitalized depending on the severity of ocular injuries and associated systemic damages. No protective eye shields were used by patients when explosion occurred. As for the injury patterns, oculoplastic injuries were seen in 71.7% patients, closed globe injuries accounted for 19.5% of the cases, open globe injuries accounted for 13.2% of the cases, and neuro-ophthalmic injuries occurred in 5.03% eyes. Patients in this study were followed ranging from 2 weeks to 5 months, which depended on the patterns and severity of the ocular injuries.

### Globe injuries

In this study, globe injuries accounted for 32.7% of the injuries. Detailed information of closed ocular injuries was noted: ocular contusion occurred in 27 cases and lamellar laceration occurred in 4 cases. There were 8 ocular contusions had zone 1,2 involved, while 19 ocular contusions had zone 3 involved. In patients suffered open globe injures, globe penetration accounted for 71.4% (*n* = 15), intraocular foreign body (IOFB) accounted for 4.8% (n = 1), and globe rupture were seen in 6 cases. Corneal lacerations were seen in 6 eyes suffering penetration, while scleral lacerations consisted the other 9 globe penetration. In globe injuries, damages to posterior structures (retina, choroid, vitreous, optic nerve) might have negative influence on the prognosis of BCVA: retinal injuries including retinal detachment, retinal tears and retinal holes occurred in 17 cases, vitreous hemorrhage occurred in 19 cases, choroidal injuries (hemorrhage, tear) occurred in 17 cases. One case with IOFB (eyelash) in this study was located in the scleral laceration which was removed during the repairment of the globe. Scleral rupture was seen in 5 globe ruptures with BCVA of NLP or LP, while corneal rupture occurred in 1 globe rupture with initial BCVA of NLP, in which extrusion of ocular contents and damages to iris, lens, ciliary body, retina, and choroid occurred to different extent.

As Tables 4 and 5 presented, both the initial visual acuity and final visual acuity were illustrated and analyzed statistically in different categories in the light of OTS. The injuries were categorized into 5 degrees based on the raw score sum ranging from 26 to 100 with the median being 70. There were 10 eyes categorized as grade1 (0–44 points) in this study, which predicted worst visual outcome as no light perception (70%) or hand move/light perception (10%). While category 2 (45–64 points) accounted for 10 eyes in our study, the visual outcome of which were improved and varied from HM to better than 20/40. Compared to the other 4 categories, eyes of category 4 (81–91 points) accounted for the most of globe injuries (*n* = 16), in which final BCVA of 70% of the eyes achieved better than 20/40.

**Table 4:** Ocular Trauma Score and likelihood of final visual acuity (*n* = 52)

Sum of the Raw points	OTS	NLP	LP/HM	1/200–19/200	20/200–20/50	≥20/40
0–44	1	73%	17%	7%	2%	1%
45–65	2	28%	26%	18%	13%	15%
66–80	3	2%	11%	15%	28%	44%
81–91	4	1%	2%	2%	21%	74%
92–100	5	0%	1%	2%	5%	92%

**Table 5:** Ocular Trauma Score and final visual acuity as followed (*n* = 45)

Sum of the Raw points	OTS	Final visual acuity				
		NLP	LP/HM	1/200–19/200	20/200–20/50	≥20/40
0–44	1	7/10 70%	1/10 10%	0%	2/10 20%	0%
45–65	2	0%	1/9 11.1%	2/9 22.2%	3/9 33.3%	3/9 33.3%
66–80	3	0%	2/12 16.7%	0%	3/12 25%	7/12 58.3%
81–91	4	0%	0%	0%	3/10 30%	7/10 70%
92–100	5	0%	0%	0%	0%	4/4100%

Globe injuries with final visual acuity followed from 14 days to 5 months post injury (*n* = 45)

Table 6 was made according to the initial BCVA and the final followed-up BCVA as documented. Statistic results showed that the final BCVA was significantly positive associated to the initial BCVA (*p* = 0.000), with 67.3% improved, 32.7% remained unchangeable. Globe rupture, retinal detachment, choroidal injury and optic nerve injury were considered to cause worse visual outcomes as final BCVA less than 20/200 with *p* < 0.05, OR = 1.088, 11.268, 11.268, 6.388, respectively. However, globe penetration was not associated with poor visual acuity (p>0.05).

**Table 6:** Ocular factors causing final visual acuity worse than 20/200

Ocular Injury	Odds Ratio	95% Confidence Interval	*P* value
Globe rupture	1.088	0.131–9.031	0.027
Retinal Detachment	11.268	1.929–65.801	0.000
Optic nerve damage	6.388	0.819–49.809	0.007
Choroidal injury	11.268	1.929–65.801	0.000
Vitreous hemorrhage			0.001
Globe penetrating			0.539
Initial Visual Acuity			0.000

Univariate statistic testing by qi-square compared final BCVA worse than 20/200 versus final BCVA better than 20/200. *P* value < 0.05 was considered statistical significance. Binary logistic regression statistic was performed followed qi-square test, OR>1 indicates negative factor for prognosis of visual acuity

Different surgical procedures were performed depending on the types of the open globe injuries, 19 ocular explorations and primary globe repairs, 2 enucleations with artificial eyes implanted as first-stage operation at 9.4 h post explosion on average ranging from 1 to 48 h. The most common second-staged surgical procedures were pars plana vitrectomy (*n* = 11) with repair of retinal detachment and silicon oil fulfilment performed on 12 eyes, with mean timing 14.7 days post explosion (range 13 ~ 16). Additionally, enucleation and orbital reconstruction were performed on 2 eyes 90 days after explosion. Two eyes were performed enucleation, for they presented as ocular atrophy after second surgery. IOFB and orbital foreign bodies were removed during the first-stage operations.

### Oculoplastic injury

In this study, adnexal injuries and orbital injuries were included in oculoplastic injuries. Eyelid laceration and contusion occurred in 101eyes, 13 of which were combined with foreign bodies (glass, dust or debris of the explosion) definitely, while 62 were uncertain. Twenty-eight eyelid injuries were combined with globe injuries, and 11 of them combined with orbital fracture. Primary lid laceration repairs and removal of foreign bodies were performed on these patients. Two eyes suffered canalicular lacerations and received an emergent canalicular laceration repairs in the operating room.

Orbital fracture accompanying globe injuries occurred on 14 eyes as documented, especially according to the radiology images we collected in the clinic, meanwhile orbital foreign bodies were presented in 4 eyes. Four orbital foreign bodies were removed by emergent surgeries including one eye with a giant orbital foreign body with a combination of ophthalmology and nasal surgeries. One orbital reconstruction was performed as second-staged surgery 90 days after the explosion.

### Neuro-ophthalmologic injury

Neuro-ophthalmologic injury discussed in this study consisted of direct (avulsion or transection caused by orbital foreign body or orbital cranial fracture or blast wave) and indirect injuries to the optic nerve. Direct optic nerve injury occurred in 8 eyes, which were accompanied with global injuries.

### Systemic comorbidities

Forthy victims suffered associated systemic injuries showing in Tables 7, 19 of which were globe injuries. Facial injury and head injury were common in the patients with ocular injuries with an incidence 11 and 6 respectively. Thirteen patients accompanied upper and lower extremity injury including fracture, muscular damage and laceration. Systemic multiple skin laceration occurred in 8 patients, and 3 severely systemic burns, 2 shocks, 3 multiple damages occurred in these patients.

**Table 7:** Associated systemic injuries occurred in victims (*n* = 40)

Facial injury (skin laceration, fracture)	11
Head injury (brain damage, skin laceration)	7
Extremity (skin laceration, fracture)	13
Trunk (skin laceration, fracture)	6
Multiple skin laceration	8
Shock	2
Severe burns	3
Multiple organ injury	3

Victims usually suffered multiple systemic injuries, hence total number of injuries listed was more than the number of victims

## Discussion

Ocular blast injury has been well documented from military experiences, however ocular injury caused by a chemical blasts on a large scale in civilian setting were seldom reported. Although eyes account for a small proportion of the bodies surface, ocular injuries are still very common in the mass-casualty incidents [3, 11, 12]. Unlike other body parts, the eyes are vulnerable to injury during a blast due to lack of protection or the presence of spectacles which can contribute to the injury.

Patterns of ocular injuries and severities were correlated to the distances to the explosion, location and the victim’s surrounding (i.e. close to the window). Most injury locations were within 500 m of the explosion site. Considering the time and resident buildings surrounding the blast, most of the patients were sleeping at home when the first small blast occurred. Some of the patients woke up and stood by the windows to observe the fire caused by the explosion when the second huge explosion occurred abruptly, which contributed to the rate of facial injuries including ocular injuries. Meanwhile, secondary projectiles (shattered glass from windows, frame or other objects) caused by the explosion accounted for the majority of the ocular injury in our study, in contrast to combat ocular injury which is mostly due to the primary blast wave. It is important to improve residents’ awareness that facing windows during an explosion may put their vision and life in danger.

In this study, BETT classification system gave guidance for classification the patterns of globe injuries and categories and details (most important ocular structure injuries) were added in order to give a comprehensive classification referred to that used in combat ocular injury [13]. Demographics of this explosion partially differed from the combat explosion ocular injury, but they were close to the recorded terrorism explosion ocular injury [12, 14].

25% globe injuries with final BCVA worse than 20/200 were found in this study, which was lower than 27% reported by Kuhn in the United States Eye Injury Registry [15]. Previous studies talked about variable risk factors potentially affecting the poor visual outcome caused by trauma, including vitreous hemorrhage, poor initial visual acuity, retinal detachment, and globe rupture or penetration, all of which involved in our study [16, 17]. Although we couldn’t test all the factors talked above due to the limation of cases of globe injury in our study, our results were aligned with those of combat ocular injuries [14]. Additionally, OTS system was well used to clarify the globe injuries to different levels correlating visual outcomes retrospectively showed in Table 2 and Table 4. Although the blast occurred abruptly and most of the resources were devoted to rescuing the survivors, initial visual acuity and other important ocular information was collected as soon as the patients were examined [18], however, there were still some patients having difficulties in cooperating with the examinations due to their unconsciousness or other vital life problems needed to be treated emergently. As for the final visual acuity, the follow-up duration varied from 14 days to 5 months depending on the severity of the trauma and patients’ compliance. The patients who had severe ocular injury had longer followed up. Victims suffered ocular blast injury and their family were eager to acknowledge their injury condition and prognosis, hitherto, it is of great significance for the ophthalmologists to perform careful eye examinations and made an exact evaluation based on the OTS.

It is common that ocular blast injury is usually associated with systemic injuries with the range from 35 to100% for the noncombat injury and higher in combat injury as 85% [19], and the rate of associated systemic injury in our study was with the range previously reported. Although vision restoration is of great significance for the patients, the primary priority is to save patients’ lives, which means ophthalmologists should be capable to make an initial and correct relative judgement about patients’ systemic condition.

In review of the ocular injuries occurring in battle, victims who had delayed treatment were more likely to have severe complications or subsequent enucleations [19]. However, none of enucleations resulted from no possibility of visual or cosmetic rehabilitation instead of delayed treatment. In contrast to the incidence of endophthalmitis in penetrating injuries as 2 ~ 7% [20], no endophthalmitis occurred in our study as the debris was sterilized by the high temperature caused by chemical explosion. Hence, the rate of eye enucleation 9.6% was lower than those reported in the battle as 16.4 [21] or noncombat explosion as 28% [22].

Second management differed after closing the globes based on injured ocular tissues, particularly when the posterior segment was involved. Although Agrawal et al. [23, 24] have reported that the timing of surgery seems to have very little effect on the final outcome, most appropriate timing for the second intervention is still under controversy due to double edged effect caused by either early or delayed vitrectomy procedure [25]. In this study, vitrectomy was performed 14.6 days post globe injuries close to 14 days as most surgeons suggested averagely aiming to remove vitreous hemorrhage and restore damaged retina or choroid as much as possible, reducing any risk of inflammation or epi-retinal membrane formation or occurrence of proliferative vitreoretinopathy, and different tamponade was used based on the severity of injuries, silicon oil was tamponaded for the patients who suffered choroidal hemorrhage or heavy vitreous hemorrhage or sub-retinal hemorrhage or retinal detachment [26, 27].

Several limitations could not be avoided in this retrospective study. Firstly, some ocular injuries were missing on account of triage to other medical resources which were included in this study, hence the sample is relatively small. On the other hand, victims were startled and urged to get medical treatments once they were sent to hospitals, hence, there was no time for the ophthalmologists to make detailed records for these outpatient ocular injuries. Due to the lack of follow-up data, no analysis of acute or chronic sequelae that the traumatic eyes are more susceptible to develop were reported [28].

## Conclusions

Consequently, it is undoubted that terrorist or non-terrorist explosion may happen to civilians in the future, and blast related ocular injuries are common injuries deserving attention. Many ophthalmologists are obligated to classify the ocular injuries, make a relatively accurate predication of recovery and carry out imperative surgeries at proper timing. We analyzed the basic characteristics of the ocular blast injury in a big fire and explosion in Tianjin with the guidance of OTS, in which globe injury presented with low initial BCVA, choroidal damage, retinal detachment, rupture and vitreous hemorrhage predict the worse prognosis. Except for intense rescue and careful examination, proper surgery should be performed correctly. In this explosion, some of the victims were hurt when they stood by the window under the curiosity, hence, more education needed to help the civilians protect themselves from unexpected explosions.

## Data Availability

The datasets used and/or analyzed in the current study are available from the corresponding author upon reasonable request.

## Abbreviations

BCVA: Best-corrected visual acuity
OTS: Ocular trauma score
BETT: Birmingham Eye Trauma Terminology
LP: Light perception
HM: Hand Motions
OR: Odds ratios
CI: Confidence intervals
IOFB: Intraocular foreign body
